# 97 Burning a Hole in Our Pockets: 12 Year Trends of Medicare Reimbursement for Burn Procedures

**DOI:** 10.1093/jbcr/irad045.070

**Published:** 2023-05-15

**Authors:** Rafael Felix Tiongco, Rena Atayeva, Iman Khan, Joseph Puthumana, C Scott Hultman, Carisa Cooney, Julie Caffrey, Cecil Qiu

**Affiliations:** Johns Hopkins University School of Medicine, Baltimore, Maryland; Johns Hopkins University School of Medicine, Baltimore, Maryland; Johns Hopkins University School of Medicine, Baltimore, Maryland; Johns Hopkins University School of Medicine, Baltimore, Maryland; Johns Hopkins University School of Medicine, Baltimore, Maryland; Johns Hopkins University School of Medicine, Baltimore, Maryland; Johns Hopkins University School of Medicine, Baltimore, Maryland; Johns Hopkins University School of Medicine, Baltimore, Maryland; Johns Hopkins University School of Medicine, Baltimore, Maryland; Johns Hopkins University School of Medicine, Baltimore, Maryland; Johns Hopkins University School of Medicine, Baltimore, Maryland; Johns Hopkins University School of Medicine, Baltimore, Maryland; Johns Hopkins University School of Medicine, Baltimore, Maryland; Johns Hopkins University School of Medicine, Baltimore, Maryland; Johns Hopkins University School of Medicine, Baltimore, Maryland; Johns Hopkins University School of Medicine, Baltimore, Maryland; Johns Hopkins University School of Medicine, Baltimore, Maryland; Johns Hopkins University School of Medicine, Baltimore, Maryland; Johns Hopkins University School of Medicine, Baltimore, Maryland; Johns Hopkins University School of Medicine, Baltimore, Maryland; Johns Hopkins University School of Medicine, Baltimore, Maryland; Johns Hopkins University School of Medicine, Baltimore, Maryland; Johns Hopkins University School of Medicine, Baltimore, Maryland; Johns Hopkins University School of Medicine, Baltimore, Maryland; Johns Hopkins University School of Medicine, Baltimore, Maryland; Johns Hopkins University School of Medicine, Baltimore, Maryland; Johns Hopkins University School of Medicine, Baltimore, Maryland; Johns Hopkins University School of Medicine, Baltimore, Maryland; Johns Hopkins University School of Medicine, Baltimore, Maryland; Johns Hopkins University School of Medicine, Baltimore, Maryland; Johns Hopkins University School of Medicine, Baltimore, Maryland; Johns Hopkins University School of Medicine, Baltimore, Maryland; Johns Hopkins University School of Medicine, Baltimore, Maryland; Johns Hopkins University School of Medicine, Baltimore, Maryland; Johns Hopkins University School of Medicine, Baltimore, Maryland; Johns Hopkins University School of Medicine, Baltimore, Maryland; Johns Hopkins University School of Medicine, Baltimore, Maryland; Johns Hopkins University School of Medicine, Baltimore, Maryland; Johns Hopkins University School of Medicine, Baltimore, Maryland; Johns Hopkins University School of Medicine, Baltimore, Maryland; Johns Hopkins University School of Medicine, Baltimore, Maryland; Johns Hopkins University School of Medicine, Baltimore, Maryland; Johns Hopkins University School of Medicine, Baltimore, Maryland; Johns Hopkins University School of Medicine, Baltimore, Maryland; Johns Hopkins University School of Medicine, Baltimore, Maryland; Johns Hopkins University School of Medicine, Baltimore, Maryland; Johns Hopkins University School of Medicine, Baltimore, Maryland; Johns Hopkins University School of Medicine, Baltimore, Maryland; Johns Hopkins University School of Medicine, Baltimore, Maryland; Johns Hopkins University School of Medicine, Baltimore, Maryland; Johns Hopkins University School of Medicine, Baltimore, Maryland; Johns Hopkins University School of Medicine, Baltimore, Maryland; Johns Hopkins University School of Medicine, Baltimore, Maryland; Johns Hopkins University School of Medicine, Baltimore, Maryland; Johns Hopkins University School of Medicine, Baltimore, Maryland; Johns Hopkins University School of Medicine, Baltimore, Maryland; Johns Hopkins University School of Medicine, Baltimore, Maryland; Johns Hopkins University School of Medicine, Baltimore, Maryland; Johns Hopkins University School of Medicine, Baltimore, Maryland; Johns Hopkins University School of Medicine, Baltimore, Maryland; Johns Hopkins University School of Medicine, Baltimore, Maryland; Johns Hopkins University School of Medicine, Baltimore, Maryland; Johns Hopkins University School of Medicine, Baltimore, Maryland; Johns Hopkins University School of Medicine, Baltimore, Maryland

## Abstract

**Introduction:**

Burn injuries cost the USA ~$976.6 million annually. Physician reimbursement has lagged despite years of lobbying by physician groups. The Centers for Medicare and Medicaid Services plan to cut physician reimbursement by 4.2% in 2023. Evaluating reimbursement data for hospital-based procedures is timely, including burn procedures. We hypothesized Medicare reimbursement trends for common burn procedures decreased from 2010-2022.

**Methods:**

We obtained pricing data from the publicly-available Medicare Physician Fee Schedule Look-Up Tool for 26 Current Procedural Terminology (CPT) codes: “Burns–preparation of wound bed” (15002-15005), “Burns–split thickness skin graft” (15100-15101 & 15120-15121), “Burns–skin substitute” (15271-15278), “Cultured epidermal autograft” (15150-15152 & 15155-15157), and “Cell Suspension Epidermal Autograft” (15110-15111 & 15115-15116). We calculated percent differences for reimbursement; compound annual growth rate (CAGR); and percent differences for work, facility, non-facility, and malpractice relative value units (RVUs). Analysis was conducted in R 4.1.2.

**Results:**

The three largest reimbursement increases were for CPT codes 15272 (24.6%), 15155 (23.7%), and 15003 (10.9%); the three largest decreases were for 15121 (-16.8%), 15120 (-6.1%), and 15275 (-5.0%). The three largest CAGR increases were for CPT codes 15272 (2.2%), 15155 (1.8%), 15274 (0.9%), and 15003 (0.9%); the three smallest were for 15121 (-1.5%), 15120 (-0.5%), and 15275 (-0.5%). Table 1 shows trends of RVUs.

**Conclusions:**

Our 12-year analysis of Medicare reimbursement trends for 26 burn procedures demonstrated an overall increase in hospital reimbursement and parallel net decrease in physician work RVUs. Reimbursements were increasingly allocated away from surgeons to facility fees and malpractice insurance. Cultured epidermal autograft to the head, neck, hands, and feet (15155) saw the largest increase in Medicare reimbursement with no change in direct physician reimbursement while split-thickness skin grafts to the head, neck, & genitals (15120 & 15121) showed decreased rates. Future work is needed to understand why these trends are occurring to advocate against physician reimbursement cuts.

**Applicability of Research to Practice:**

Studies contributing to price transparency allow stakeholders to focus on why burn surgeons are receiving lower reimbursements.

